# No Cutting Corners: The Effect of Parental Involvement on Youth Basketball Players in Israel

**DOI:** 10.3389/fpsyg.2020.607000

**Published:** 2020-11-16

**Authors:** Assaf Lev, Adi Bichman, Avi Moyal, Shmulik Brenner, Nir Fass, Ella Been

**Affiliations:** ^1^Department of Sports Therapy, Faculty of Health Professions, Ono Academic College, Kiryat Ono, Tel Aviv-Yafo, Israel; ^2^School of Education, Ono Academic College, Kiryat Ono, Israel; ^3^Department of Anatomy and Anthropology, Sackler Faculty of Medicine, Tel Aviv University, Israel

**Keywords:** parent–child relationship, basketball, competitive sports, questionnaires, child’s satisfaction

## Abstract

This paper explores the nature of parental involvement in youth basketball in Israel with regard to parenting style and in the context of dilemmas and ethical issues. It is well established that parental involvement in their child’s sporting activity has vast implications on the child’s motivation and enjoyment. With reference to Israeli society, only a few studies have focused on this subject. In order to address this lacuna, we used two questionnaires, given to 173 youth basketball players (child questionnaire) and their parents (parent questionnaire). Key findings illustrate three main themes. First, a higher level of satisfaction and contentment among basketball players whose parents demonstrated greater involvement; second, that parental emotional involvement is the most important variable for young athletes’ satisfaction; and finally, differences in gender roles reveal that fathers are more involved with logistics, while mothers are more dominant in emotional involvement. Moreover, the findings demonstrate that parents should mainly place emphasis on emotional involvement. However, we suggest that parents do not bypass logistical care as this may create opportunities for greater emotional support and therefore greater child satisfaction.

## Introduction

Over the course of the last two generations, there has been growing awareness by parents relating to their child’s development in sports (Wheeler and Green, 2014; Harwood et al., 2019; Knight, 2019). While exploring this shift, one must take into account cultural differences (Cheung and Pomerantz, 2011; Knight et al., 2017), parent–teacher or parent–coach factors, as well as historical, demographic, political, and economic issues (Hornby and Lafaele, 2011; Harwood et al., 2019). Parents have become more involved in their child’s sports activities, which has implications on the parent–child relationship and the complex socialization process that a child goes through (Dorsch et al., 2015). The reasons parents decide to become involved in their child’s sport activity are varied. Some regard it as a model of good parenting, while others use it as a way to develop intimacy with their child (Stefansen et al., 2018). These dynamics can create conflicts such as child frustration due to the pressure of high parental expectations or a feeling of disappointment by the parent if the child is not interested in sport (Sorkkila et al., 2017). The parents and the child may have different perspectives on parental involvement, which can lead to discord. While the parent regards the various forms of more active engagement in the child’s sport as encouraging and good parenting, the child may view this engagement as criticism and pressure (Elliott and Drummond, 2013). Considering the social nature of competitive sport and the emphasis placed on achievement in society, the relationship between parents and their child becomes a critical element in the healthy development of young athletes today (Welk et al., 2003).

Much has been written about the positive relationship between active parents and the good physical activity habits of their child, and the negative effect of less active parents (Cleland et al., 2005; Eriksson et al., 2008; Tate et al., 2015; Folle et al., 2018; Rodrigues et al., 2018). However, as not all parents are the same, it is important to recognize the differing attitudes among them and to consider this diversity in an attempt to create more tailored interventions and educational materials (Knight et al., 2017).

### Parental Influence on the Youth Player

Parents are usually involved in their child’s sport activity from a young age and have an influence on the child’s initial involvement and long-term participation in sport (Fraser-Thomas and Côté, 2009). This involvement can have a positive or negative influence on a child’s sporting development, experiences, and emotions (Knight and Holt, 2014). The importance of parents’ behavior for their child’s enjoyment and motivation in sports is broadly described by the Sánchez-Miguel et al. (2013) study, which demonstrates a positive relationship between parental support of the sport and a player’s enjoyment and motivation. Moreover, it suggests that in Spanish players who perceive more pressure from their parents, there is a positive association with motivation and a negative association with enjoyment. Further evidence is found in professional female gymnasts in Brazil (Nunomura and Oliveira, 2013), where parental support enables and greatly influences a child’s entrance into sports, their access to the practice of a sport, their level of participation, their degree of involvement, and their physical and emotional well-being. However, when this support is perceived negatively, it can result in stress, conflicts between parents and the child, burnout, and eventual dropout. This negative influence is observed in a study, which examines the relationship of young Australian football players and their parents after the match ended (Elliott and Drummond, 2013). While the parents perceive their involvement, including verbally assessing the child’s performance as useful, the child regards it as criticism or negativity. The end result is a negative effect on the child’s performance—if a child receives criticism for dropping a mark (catch), they may deliberately avoid future attempts to mark the ball. In order to optimize parental involvement, it is important to understand the individual child’s preferences and needs in sport and the gap between the parents’ and the child’s perspectives (Knight et al., 2010; Knight and Holt, 2013; Knight and Holt, 2014; Knight, 2019). It is also relevant to highlight the area of sport-based positive youth-development (PYD) in the context of parental involvement (Holt and Sehn, 2008; Harwood et al., 2019; Holt et al., 2020). In this regard, Harwood et al. (2019) stressed that sport parents’ influence on their child’s psychosocial development is less explored. Moreover, they highlight the importance of improving the study of parental involvement in relation to the experience and development of young athletes. In this context, the current study aims to address this issue by shining a spotlight on parental involvement and the child’s satisfaction and contentment.

### Israeli Parental Involvement in Sporting Activity

Parental involvement in their child’s sports is a global phenomenon and, for this reason, should be examined within the sociocultural context. Ultimately, the linkage between parental involvement and its effect on a child’s subsequent perceptions regarding their sport’s field is influenced by broad sociocultural characteristics (Knight et al., 2017; Harwood et al., 2019; Holt et al., 2020). For example, Kang et al. (2015) find significant correlation between the self-esteem of Korean high school athletes and a level of trust and communication with their parents. Knight et al. (2016) show that parental involvement among American and British parents depends on various factors such as the type of sporting activity, observance of other parents, previous experience and knowledge, set of expectations, and social values. McMahon and Penney (2015) find that parents of Australian amateur swimmers tend to discipline their child when they do not meet their ideals. Côté (1999) and Wall et al. (2019) examine parental involvement in Canadian youth sport. While Côté (1999) emphasizes the importance of the family in the context of the development of young athletes, Wall et al. (2019) highlights the complexity of the relationship between the coaches in figure-skating and the athletes’ mothers. However, very little has been written in reference to parental involvement in their child’s sporting activity in Israel. The studies that do exist mainly focus on soccer, the most popular sport in Israel. For example, Gershgoren et al. (2011) show how parental feedback influences the child’s motivation and performance. In their study, they find that feedback, which focuses on abstract, general compliments intending to increase the child’s sense of self-worth results in the child’s perception of their performance to increase, while their actual skills on the field remain level. On the other hand, parental feedback based on specific, concrete actions by the child on the field during the course of the game does not increase the young player’s ego and improves performance over time. In a different study, Gindi et al. (2016) examine the way youth soccer coaches cope with parental involvement. They show that coaches do acknowledge the positive aspects of parental involvement, mainly logistical (e.g., driving their kids to practices), however, the researchers indicate that this involvement results in increased parental attempts of involvement in the coaches’ professional decisions. As a result, coaches find themselves frustrated and, faced with growing pressure, increasingly compliant with the parents’ demands. Muchtar et al. (2013) emphasize the importance of familial support on the development of young professional tennis players in Israel. Their findings align with multiple studies, which examine this issue (see: Gould et al., 2006; Knight et al., 2010). Broadly speaking, in reference to the Israeli culture, Tesler et al. (2019) claims that the level of physical activity, not necessarily in team sports, among Israeli youth is influenced significantly by family involvement. According to the researchers, parental involvement that includes an active sporting engagement increases the child’s level of physical activity and results in a general embrace of a healthy lifestyle. These findings can be relevant given that 28.8% of Israeli adolescents are considered overweight on the BMI (body mass index) scale. Therefore, providing parents with this data can help contribute to their success in implementing long term well-being for their child.

It is important to note that in the Israeli context, one predominant factor is mandatory army service, which plays a significant role in shaping citizens’ mentality (Sasson-Levy and Lomsky-Feder, 2018) and influences leisure time physical activity and gender inequality (Lev and Hertzog, 2017).

### Gender and Family Roles in Parental Involvement

As parental influence is clearly significant, whether in creating the optimal support structure or in role modeling, it is important to reflect on the differences between parents. This distinction is made in the Palomo-Nieto et al. (2011) study, which identifies the different roles a mother and a father play in their child’s sport activity. The mother occupies a much more encouraging role and gives unconditional support; this support takes the form of emotional assistance, regardless of her child’s actual sport performances, and is a result of the strong mother–child bond, which exists from early childhood. The father, on the other hand, may have a negative emotional impact on the child as he often dominates the role of second coach, which may lead to unnecessary pressure on the child. It should be noted that these assumptions must consider the child’s age, as the mother’s sociological and emotional influence is predominant mostly in childhood (Chan et al., 2012). Although the father’s emotional influence may lead to burnout and frustration by the child, their level of sport activity is related to positive support (Eriksson et al., 2008). In his study, Eriksson provides evidence to the notion that athletic competence is related to the association a child has of sports based on parental athletic activity and that the father’s participation in sport is of particular importance. In other words, a father who is a sporting role model can contribute to his child’s self-determined motivation for sport. However, current research indicates that both mothers’ and fathers’ physical activity are influential for a child’s sport participation, and that the influence of parental modeling appears to be stronger in parent–child pairs of the same gender (Cleland et al., 2005; Rodrigues et al., 2018). The family context is further developed in a study investigating young Brazilian female basketball players (Folle et al., 2018), which finds that families for whom practicing basketball plays an important role in their routine, serve to motivate the child to persevere in the basketball club. This reinforces the correlation between autonomy support and child motivation, and asserts that active, involved parents contribute to the construction of an environment that is favorable to the positive development of athletes. This involvement or participation in their child’s sport routine can be manifested either by presence at matches, emotional encouragement, financial support, or even moderate pressure during training sessions and matches (ibid).

In light of the literature reviewed and given the paucity of research on Israeli parents’ involvement, the aim of this study is to focus on the psychosociological aspects of parental involvement in their child’s basketball performance.

## Methodology

A total of 173 couples of parent and child answered the questionnaires. Out of the 173 parents, 51.4% are males and 48.6% are females, of an average age of 45.9 (*SD* = 6.4) years. Out of the 173 children, 78% are boys and 22% girls of an average age 13.7 (*SD* = 1.7) years, all of whom are active basketball players practicing an average of 5–10 h per week. The participants hail from various regions throughout Israel ranging from small villages to large cities.

*Questionnaire*: In this study, the researchers used two questionnaires—“parent questionnaire” and “child questionnaire” (Lev et al., 2021; Supplementary Material). Each questionnaire consisted of 22 valid and reliable questions regarding parents’ involvement—logistical and discourse, parental expectations and contentment (parent questionnaire) and child contentment, expectation, motivation, and parental discourse (child questionnaire). Out of each questionnaire, the researchers chose the relevant items to examine for this study. Each item stood independently and was found to be reliable and valid (Lev et al., 2021). Each question was scored on a five-point Likert-type scale ranging from strongly disagree (1) to strongly agree (5).

The researchers are interested, however, in a more encompassing measure that demonstrates three main themes: parental logistical involvement (items P5,6,17–19 from parents’ questionnaire); parental emotional involvement (items P7,8,11 from parents’ questionnaire); and finally, the child’s satisfaction/contentment (items C4–6,21,22 from child’s questionnaire).

Statistical analysis was performed using SPSS (v.24). Data were expressed as means ± standard deviation (*SD*). Student *t-*test was used to compare between the results of mothers and fathers. Cronbach’s α was used to determine internal consistency for the subscale scores “logistic involvement” and “emotional involvement” for parental questions and for “child’s satisfaction.” Mean scores were computed for each of those subscales. Pearson’s correlation analysis was used to determine the relationship between parents’ and child’s answers. Multiple regression was used to identify which kind of involvement is the best for describing the variation in child’s satisfaction. A value of *p* < 0.05 was considered statistically significant.

## Results

### Descriptive Statistics

The average and standard deviation of the parents’ answers are seen in Table 1 and those of the children in Table 2. Israeli parents show high involvement (average score > 3; Table 1) with their child’s sports activity when it comes to shuttling to practices and games, attending games, and spending time talking with their child about their participation in practices and games, performance, and feelings (items P6,7,8,11,18). On the other hand, they show moderate involvement (1 < average score < 3; Table 1) with the coaching staff, game referees, attending practices, and filming their child during practices/games (items P5,15,16,17,19). Both mothers and fathers show high logistical and emotional involvement with their child’s sports activity, but there are some substantial differences between the two. While fathers show higher involvement with the logistical side such as attending practices or games, and talking to the referees or their child during the game (items P15–17,19), mothers show higher involvement with the emotional side, especially talking with their child about their emotions (item P11).

**TABLE 1:** Descriptive statistics for parental (P) answers and the significance of comparison between mothers and fathers (*p* value).

**Question/item**	**All x̄ ± *SD***	**Male x̄ ± *SD***	**Female x̄ ± *SD***	***p* value Male/female**
P5. How involved are you with your child’s coaching staff?	2.99 ± 1.29	2.82 ± 1.21	3.18 ± 1.35	0.068
P6. How involved are you with shuttles of your child to practice and games?	3.87 ± 1.22	3.79 ± 1.20	3.95 ± 1.24	ns
P7. To what extent is there a dialog between you and your child regarding practice and games?	3.97 ± 1.10	3.88 ± 1.12	4.07 ± 1.08	ns
P8. To what extent is there a dialog between you and your child regarding his performance as a basketball player?	3.79 ± 1.19	3.70 ± 1.24	3.89 ± 1.13	ns
P11. To what extent do you talk to your child about feelings (joy, stress, anxiety…) that your child is experiencing on the basketball court?	3.78 ± 1.07	3.53 ± 1.12	4.04 ± 0.94	**0.002**
P15. To what extent do you have a dialog with the referees or the scoring table during official games?	1.77 ± 1.15	1.97 ± 1.21	1.57 ± 1.04	**0.024**
P16. To what extent is there communication between you and your child during games?	2.25 ± 1.23	2.49 ± 1.26	1.99 ± 1.14	**0.07**
P17. How often do you film team games/practices?	2.66 ± 1.38	2.54 ± 1.31	2.79 ± 1.45	ns
P18. How often do you come to games during the year?	3.84 ± 1.18	4.02 ± 1.01	3.64 ± 1.32	**0.035**
P19. How often do you come to practices during the year?	2.12 ± 1.20	2.37 ± 1.24	1.86 ± 1.11	**0.005**
Parental logistic involvement	3.10 ± 0.84	3.11 ± 0.78	3.08 ± 0.91	ns
Parental emotional involvement	3.84 ± 0.93	3.70 ± 0.94	3.99 ± 0.93	**0.043**

*ns, non-significant; bold, *p* < 0.05.*

**TABLE 2:** Descriptive statistics for young basketball players’ (C) answers.

**Question/item**	***n***	**Average**	****SD****
C4. To what extent are you satisfied with your social integration in the team?	173	4.45	0.78
C5. How satisfied are you with your participation in practice?	173	4.39	0.79
C6. How satisfied are you with your participation in games?	172	4.06	1.07
C7. To what extent do you invest in improving your athletic abilities beyond the team practice framework?	172	4.02	0.94
C8. What is your parents’ involvement in practice and games shuttles?	173	3.90	1.19
C9. How satisfied are you with your parents’ involvements in practice and games shuttles?	173	4.32	1.04
C11. To what extent do you estimate that your parents’ involvement has an impact on your athletic performance?	171	3.63	1.12
C12. To what extent would you like your parents to come to your games?	172	4.36	0.92
C13. To what extent would you like your parents to come to your practices?	172	2.77	1.35
C18. To what extent do your parents come to games during the regular season?	173	3.98	1.11
C19. To what extent do your parents come to practices during the regular season?	173	2.02	1.24
C20. To what extent do you think your performances influence your parents’ mood?	173	3.22	1.32
C21. How much do you enjoy playing basketball?	173	4.74	0.58
C22. How satisfied are you with your sporting performances?	173	4.10	0.82
Child overall satisfaction	173	4.35	0.61

Overall, the children in this study report very high satisfaction and enjoyment (average score > 4) regarding participation in their sports activity, their social integration, and their sporting performances (Table 2; items C4–6,21,22), and they invest in improving their athletic abilities beyond the team practice framework (item C7). Children also express that they want their parents to show high involvement in their sports specifically when it comes to shuttling them to practices/games and to attending games (Table 2; items C9,12,13). Additionally, children estimate that their parents’ involvement has an impact on their athletic performance (item C11), and they believe that their sports performances influence their parents’ mood (item C20).

### Correlations

The researchers performed Pearson correlations coefficient to explore interaction between the parents’ and the children’s answers (Table 3). In general, higher involvement of the parents in their child’s basketball activity (items P5–19) positively correlated with the child’s answers regarding their satisfaction (items C4,5,6,9,21,22). More specifically, the researchers found weak but significant correlations between parents’ involvement with the coaching team (item P5) and the child’s satisfaction (0.138 < *r* < 0.256); weak but significant correlations between parents’ involvement with shuttling the child to practices and games (item P6) and the child’s satisfaction (0.176 < *r* < 0.353) in all but two questions (items C6,21); weak to moderate correlations (0.152 < *r* < 0.415) between parental dialog with their child (items P7,8,11) and their child’s satisfaction, with the exception of C21; weak but significant correlations between parents’ involvement with game referees (item P15) and the child’s satisfaction (0.159 < *r* < 0.300) in all but two questions (items C5,9); weak significant correlations between parents’ interaction with the child during the game (item P16) and the child’s satisfaction are found only in two questions (items C6,22); weak but significant correlations between parents’ involvement with filming and photographing the game (item P17) and the child’s satisfaction (0.143 < *r* < 0.243) in all but one of the child’s questions (item C9); weak but significant correlations between parents’ attending the child’s games (item P18) and all of the measures of the child’s satisfaction (0.164 < *r* < 0.277). There was no correlation between how often parents attended the child’s practices (item P19) and the child’s satisfaction.

**TABLE 3:** Matrix of correlations between parental answers (P) and young basketball players’ answers (C).

**Question**	**P5**	**P6**	**P7**	**P8**	**P11**	**P15**	**P16**	**P17**	**P18**	**P19**
C4	0.181**	0.191**	0.306**	0.316**	0.344**	0.159*	0.070	0.143*	0.273**	0.047
C5	0.256**	0.210**	0.348**	0.415**	0.323**	0.079	0.095	0.243**	0.277**	-0.032
C6	0.215**	0.024	0.278**	0.310**	0.318**	0.181**	0.139*	0.160*	0.214**	0.085
C7	0.120	0.069	0.156*	0.227**	0.156*	0.196**	0.071	0.039	0.139*	0.164*
C8	0.204**	0.536**	0.220**	0.237**	0.224**	-0.045	0.028	0.141*	0.290**	0.124
C9	0.165*	0.353**	0.325**	0.393**	0.304**	-0.100	-0.050	0.115	0.164*	0.021
C11	0.230**	0.157*	0.272**	0.350**	0.245**	0.233**	0.170*	0.195**	0.199**	0.060
C12	0.223**	0.417**	0.313**	0.332**	0.308**	0.224**	0.219**	0.306**	0.449**	0.098
C13	0.159*	0.078	0.058	0.063	0.029	0.260**	0.197**	0.162*	0.144*	0.358**
C18	0.332**	0.419**	0.261**	0.352**	0.287**	0.160*	0.200**	0.306**	0.651**	0.187**
C19	0.269**	0.029	0.039	0.102	0.021	0.278**	0.187**	0.182**	0.209**	0.650**
C20	0.147*	0.015	0.082	0.161*	0.057	0.356**	0.286**	0.184**	0.164*	0.112
C21	0.138*	0.240**	0.358**	0.482**	0.295**	-0.007	0.023	0.075	0.219**	0.013
C22	0.138*	0.176*	0.191**	0.190**	0.152*	0.300**	0.216**	0.225**	0.232**	0.123

*p < 0.05, **p < 0.01.

The researchers divided parental involvement into “logistical involvement,” which was the average score for parental items 5, 6, 17–19, and to “emotional involvement,” which was the average score for parental items 7, 8, and 11 (Table 1). They found high internal consistency between the questions that constituted each of the parental involvement themes (Cronbach’s α = 0.688). They also created a new theme regarding a child’s overall satisfaction, which was the average score for items 4–6, 21, 22. They found high internal consistency between the five questions that constituted the child’s satisfaction theme (Cronbach’s α = 0.788). Correlation analysis revealed significant, moderate correlation between parental logistical and emotional involvement (*r* = 0.540), indicating that parents who spent more time and effort in logistics relating to their child’s sports activity also showed more emotional involvement. The researchers also found significant positive correlations between a child’s satisfaction and parental logistical and emotional involvement (*r* = 0.321, *r* = 0.479, respectively).

### Mediation Effect

Multiple linear regression analysis was used to develop a model for predicting the child’s satisfaction from the parents’ logistical involvement and the parents’ emotional involvement. The two-predictor model was able to account for 48.4% of the variance in the child’s satisfaction, *F*(2,170) = 26.053, *p* < 0.001. As mentioned previously, each of the predictor variables had a significant (*p* < 0.001) zero-order correlation with the child’s satisfaction, but only the parental emotional involvement had significant (*p* < 0.001) partial effects in the full model. Therefore, parental emotional involvement was the most important variable for a child’s satisfaction. The zero-order correlations between parental logistical involvement and a child’s satisfaction showed that there was a statistically significant, moderate, positive correlation (*r* = 0.321, *p* < 0.001), but while controlling for parental emotional involvement, the correlation was not statistically significant (*r* = 0.084, *p* > 0.05).

## Discussion

The aim of this paper is to shed light on parents’ level of involvement with their child’s sporting activity in Israel. It is generally agreed that parental involvement has vast implications on a child’s motivation and enjoyment, however, as many researchers have suggested (Cheung and Pomerantz, 2011; Knight et al., 2017; Harwood et al., 2019; Holt et al., 2020) this phenomenon has to be comprehended in the sociocultural context. Thus, this paper makes a unique contribution by exploring the nature of parental involvement on youth basketball in Israel when it comes to parenting style in the context of dilemmas and ethical issues.

The findings reveal that Israeli parents invest great time in their child’s sporting activity; more specifically, parents invest time in shuttling their child to practices and games and in talking with their child about their physical and emotional well-being and the child’s performances at practices and games. These findings are in line with the recent Furusa et al. (2020) study from Britain, which demonstrates the child’s desire for their parents to not only provide tangible support but also to be actively engaged in the discussions about their sporting performances. However, the researchers find that while regularly attending games, Israeli parents are only semi-attending practices, documenting their child’s performances on camera and talking with professional staff. This finding contradicts the Gindi et al. (2016) study, which examines the way youth soccer coaches cope with parental involvement. While the researchers demonstrate a high level of logistical engagement among the Israeli soccer parents in their study, leading to more parental interference with coaches during practices, in the current study, the Israeli basketball parents are less engaged in the child’s practices altogether. This situation may present an ethical dilemma to Israeli parents who question whether to increase their involvement or allow their child greater autonomy (Harwood et al., 2019). There are various autonomous domains, which may be allowed for adolescents of a certain age, such as logistical independence (driving oneself to practices/games), boundaries during games (allowing coaches and referees to make decisions without interference, and not directing their child during the course of the game), and encouraging the child to assert their independence (lack of need of “helicopter parenting”). The findings of the current study show that the level of parental, logistical involvement in Israel is high (P6 > 3) contrasted with their involvement with coaches and during games (P5,15,16 < 3). The children in this study display high levels of satisfaction and enjoyment from the sport; therefore, the researchers conclude that the domain in which parents are best to provide greater autonomy to their child lies in the area of non-interference with sporting staff. Thus, as opposed to the parents of youth soccer players (Gindi et al., 2016), parents of adolescent basketball players should consider not becoming overly intrusive with their child’s coaches. This aligns with Gould et al. (2008) and Lauer et al. (2010) who stress that when American parents interfere with coaches, it can result in pressure and anxiety for the child. With regard to the differences between the parents of basketball players versus those of soccer players, the researchers suggest the possibility that due to soccer’s popularity over basketball in Israel, soccer parents are more inclined to attend and be actively involved in both practices and games.

The current study finds a higher level of satisfaction and contentment among youth basketball players whose parents demonstrate higher levels of involvement, and vice versa. Put simply, the more parents are involved in their child’s sporting activity, the higher the child’s contentment and satisfaction with the sport. These findings support the Sánchez-Miguel et al. (2013) study, which demonstrates a positive relationship between parental support of the sport and a player’s enjoyment and motivation among youth Spanish athletes. However, the findings of this study seem to contradict scholars who suggest that parental support acts as a stressor that diminishes the athlete’s physical and emotional well-being (Elliott and Drummond, 2013; Knight and Holt, 2014) among Australian and British athletes. This can be partly explained given that Israeli culture values tight-knit families. Although Israeli families are undergoing changes as part of the postmodern trend of adolescents seeking greater autonomy at a younger age, they still have relative solidarity and emotional closeness (Peer, 2014; Ben-Nun and Tene, 2018).

Further, in the current study, the researchers find a strong correlation between emotional parental involvement and their child’s satisfaction, while logistical parental involvement contributes to a lesser extent. This is not to say that the latter is not important or is negligible. After all, there is vast agreement among scholars that parental logistical support demonstrates to the child that their parents value their sporting activity and thus enhances feelings of enjoyment, competence, motivation, and persistence (Knight et al., 2010; Harwood et al., 2019). However, this study emphasizes that logistical support cannot stand alone; it must include emotional support. Although shuttling their child to practices and games can be perceived as strictly technical, it should be stressed that parents do not perform as “taxi drivers” providing impartial engagement. On the contrary, driving their child constitutes active engagement, which can facilitate profound dialog between parents and their child before and after practices/games. Elliott and Drummond (2017) find that verbal comments, for example, those articulated in the car in the after-sport experience are interpreted as negative and thus weaken the enjoyment of sporting experiences for the child. As demonstrated in this particular research, the findings are not in line with those of Elliott and Drummond. The results of the study indicate that verbal comments by the parents in transit during the after-sport experience have the potential to increase a young athlete’s satisfaction in the game. The implications are noteworthy. First, this study highlights the significance of being involved in a child’s sporting activity not only emotionally but also logistically. Hence, Israeli parents who send their child to practices/games via taxi or another form of public transportation do not impart the emotional component, thus missing the opportunity for emotional engagement with their child. Parents may be torn with the question of the appropriate level of involvement and whether what they are doing is enough for their child’s satisfaction and well-being. In modern times, busy parents often face a dilemma when they feel they must choose either logistical or emotional involvement with their child based on limits on their time due to various other responsibilities such as their professional careers. The researchers highly recommend that parents not bypass logistical support opportunities as these lead to an enhancement of a child’s contentment and satisfaction in their sporting activity. Thus, the results indicate that the role of the parents in not cutting corners, in other words, providing both emotional and logistical support contributes to the notion of sport-based PYD. Moreover, the analysis of this study also shows a gender variation with regard to the nature of parental involvement. While both parents play a significant role in their child’s sporting activity, the findings reveal that fathers are more involved with logistical aspects (such as attending games and practices and speaking with the coach), whereas mothers are more involved with providing emotional support. In this respect, mothers constitute a “sounding board,” which helps a child express their emotions. This finding is in line with the Palomo-Nieto et al. (2011) study, which stresses that Spanish mothers offer emotional assistance, assuming a much more encouraging role and giving unconditional support. Nevertheless, the current study’s findings contradict the (Palomo-Nieto et al., 2011) conclusion that fathers have a negative emotional impact on the child as they often dominate the role of “second coach” leading to unnecessary pressure on the child. As mentioned above, in this study, a child perceives emotional and logistical involvement from both parents as supportive, which increases their contentment, satisfaction, enjoyment, and motivation with the sport. In order to understand the differing gender roles, the researchers believe it is important to emphasize the discourse of militarization in Israeli society, due to the gender separation of certain units within the Israeli army, which implicitly reinforces domestic roles (Sasson-Levy and Lomsky-Feder, 2018). The clear gender divide in Israel among parents is not surprising due to mandatory army service resulting in gender inequality, which pervades everyday life (ibid). This reality can also take place in Israeli sport leisure activities (see: Lev and Hertzog, 2017; Hertzog and Lev, 2019).

This study examines numerous youth basketball teams in Israel, however, it concentrates mainly on the adolescent, male, and Jewish population. In some regard, their experiences may differ from adolescents of other genders, cultures, and religions. Therefore, the study is limited due to its focus on only one specific ethnic group and only a specific sport. A further study could include research that follows parents’ level of involvement with their child’s sporting activity among both genders within different sports and different ethnic groups. It is important to stress that this study emphasizes parental involvement at a specific time and, therefore, does not provide any information regarding the previous parent–child relationship. Moreover, while this study is based on a quantitative approach, a further study on parents’ level of involvement with their child’s sporting activity in Israel using a qualitative approach, such as semi-structured interviews and observations, is suggested. This has the potential to encourage participants to provide their own unique context for events, feelings, and behaviors (see Lev, 2019, 2020).

Further, given that Israel recently placed second among European countries in adolescent BMI (body mass index) rankings with 28.8% of youth overweight, this study can contribute to encouraging youth to continue with sporting activity by helping parents understand the importance of their interactions with their child regarding sports. The paper constitutes an effective tool to aid Israeli parents in comprehending the value of their approach and how this approach might lead to a child’s increased satisfaction and enjoyment with sporting activity, therefore increasing adolescent health. Finally, as little has been written in reference to the parent/child relationship in youth basketball in Israel, this paper addresses the lacuna of literature regarding parental involvement in sports. Harwood et al. (2019) remind us that there is a certain homogeneity in the literature in reference to the “best” parenting style within youth sport. In this regard, this study also contributes to the scope of parental involvement in sport by shedding light on Israeli youth basketball players.

## Data Availability Statement

The raw data supporting the conclusion of this article will be made available by the authors, without undue reservation.

## Ethics Statement

The studies involving human participants were reviewed and approved by Ono Academic College, Ethics Committee. Written informed consent to participate in this study was provided by the participants’ legal guardian/next of kin.

## Author Contributions

AL conceived the presented idea, assembled the questionnaires, wrote the manuscript, analyzed the results, and supervised the project. AB assembled the questionnaires and collected the data. AM, SB, and NF assembled the questionnaires. EB performed the analysis, assembled questionnaires, wrote the manuscript, and supervised the project. All authors contributed to the article and approved the submitted version.

## Conflict of Interest

The authors declare that the research was conducted in the absence of any commercial or financial relationships that could be construed as a potential conflict of interest.
