# Supplementary material for: No Cutting Corners: The Effect of Parental Involvement on Youth Basketball Players in Israel
Source: Front Psychol. 2020 Nov 16;11:607000. doi: 10.3389/fpsyg.2020.607000 (PMC7701243; doi:10.3389/fpsyg.2020.607000)
Supplement: Supplementary file 1 [file Data_Sheet_1.pdf]

Supplementary material

Both parent and child questionnaires presented here were originally written in Hebrew and have been translated to English.

Parent questionnaire

Id:

Consent: Y/N

Child Consent: Y/N

Age:

Sex:

Marital status:

In what city do you live?

In what grade is your child?

What is your child's jersey number?

Have you been involved in competitive sports in the past?

Are you involved in competitive sports today?

How many hours per week do you spend on sporting activity? 0, 1-2, 3-5, 5-10 more than 10

Question number	Question	1	2	3	4	5
		Not at all				To a large extent
1	To what extent are you satisfied with your child's social integration with the team?					
2	How satisfied are you with your child's participation in practices?					
3	How satisfied are you with your child's participation in games?					
4	To what extent are you satisfied with your child's investment in improving his/her athletic abilities beyond the team practice framework?					
5	How involved are you with your child's coaching staff?					
6	How involved are you with the shuttling of your child to and from practices and games?					
7	To what extent is there a dialogue between you and your child regarding practices and games?					

8	To what extent is there a dialogue between you and your child regarding his/her performance as a basketball player?					
9	How involved are you in maintaining a healthy lifestyle (diet, sleep, etc.) with your child?					
10	To what extent do you think your child experiences stress/anxiety during games?					
11	To what extent do you talk to your child about feelings (joy, stress, anxiety, etc.) that he/she is experiencing on the basketball court?					
12	To what extent do you expect your child to become a professional basketball player?					
13	To what extent do you set a personal example for your child in dealing with difficulties on court?					
14	To what extent does your child aspire to be a professional basketball player in the future?					
15	To what extent do you have a dialogue with the referees or the scoring table during official games?					
16	To what extent is there communication between you and your child during games?					
17	How often do you film team games/practices?					
18	How often do you come to games during the year?					
19	How often do you come to practices during the year?					
20	How satisfied are you with your child's athletic performance?					
21	To what extent do you estimate that your involvement and participation as a parent has an impact on your child's athletic performance?					
22	To what extent do you think your child enjoys playing basketball?					

Child questionnaire

Id:

Consent: Y/N

Age:

Sex:

In what grade are you?

In what city do you live?

What is your jersey number?

How many hours a week do you spend on sport? 0, 1-2, 3-5, 5-10 more than 10

Question number	Question	1	2	3	4	5
		Not at all				To a large extent
1	To what extent is there a dialogue between you and your parents regarding your practices and games?					
2	To what extent is there a dialogue between you and your parents regarding your performance as a basketball player on the team?					
3	To what extent is there a dialogue between you and your parents regarding maintaining a healthy lifestyle (nutrition, sleep, etc.)?					
4	To what extent are you satisfied with your social integration in the team?					
5	How satisfied are you with your participation in practices?					
6	How satisfied are you with your participation in games?					
7	To what extent do you invest in improving your athletic abilities beyond the team practice framework?					
8	What is your parents' involvement in shuttling to and from practices and games?					
9	How satisfied are you with your parents' involvement in shuttling to and from practices and games?					
10	To what extent do you talk to your parents about the emotions (joy, stress, anxiety, etc.) that you experience on court?					
11	To what extent do you estimate that your parents' involvement has an impact on your athletic performance?					
12	To what extent would you like your parents to come to your games?					
13	To what extent would you like your parents to come to your practices?					
14	To what extent does your mother serve as a role model for you in dealing with challenges on court?					
15	To what extent does your father serve as a role model for you in dealing with challenges on court?					
16	To what extent is there communication between you and your parents during games?					
17	To what extent is there communication between you and your parents during practices?					

18	To what extent do your parents come to games during the regular season?					
19	To what extent do your parents come to practices during the regular season?					
20	To what extent do you think your performance influences your parents' moods?					
21	How much do you enjoy playing basketball?					
22	How satisfied are you with your sporting performance?					
